# Influenza surveillance in Western Turkey in the era of quadrivalent vaccines: A 2003–2016 retrospective analysis

**DOI:** 10.1080/21645515.2018.1452577

**Published:** 2018-04-25

**Authors:** Sevim Meşe, Aysun Uyanik, Alev Özakay, Serdar Öztürk, Selim Badur

**Affiliations:** a National Influenza Reference Laboratory, Istanbul University, Istanbul, Turkey; b GSK, Istanbul, Turkey

**Keywords:** Distribution, influenza A and B, quadrivalent subunit influenza vaccines, surveillance, turkey, vaccination

## Abstract

Human influenza is predominantly caused by influenza A virus (IAV) – A/H1N1 and/or A/H3N2 – and influenza B virus (IBV) – B/Victoria and/or B/Yamagata, which co-circulate each season. Influenza surveillance provides important information on seasonal disease burden and circulation, and vaccine content for the following season. To study the circulating influenza subtypes/lineages in western Turkey. Community-based sentinel surveillance results during 2003–2016 (weeks 40–20 each season; but week 21, 2009 through week 20, 2010 during the pandemic) were analyzed. Nasal/nasopharyngeal swabs from patients with influenza-like illness were tested for influenza virus and characterized as A/H1N1, A/H3N2, or IBV. A subset of IBV samples was further characterized as B/Victoria or B/Yamagata. Among 14,429 specimens (9,766 collected during interpandemic influenza seasons; 4,663 during the 2009–2010 pandemic), 3,927 (27.2%) were positive. Excluding the pandemic year (2009–2010), 645 (27.4%) samples were characterized as A/H1N1 or A/H1N1/pdm09, 958 (40.7%) as A/H3N2, and 752 (31.9%) as IBV, but the dominant subtype/lineage varied widely each season. During the pandemic year (2009–2010), 98.3% of cases were A/H1N1/pdm09. IBV accounted for 0–60.2% of positive samples each season. The IBV lineages in circulation matched the vaccine IBV lineage >50% in six seasons and <50% in four seasons; with an overall mismatch of 49.7%. IBV cases tended to peak later than IAV cases within seasons. These results have important implications for vaccine composition and optimal vaccination timing. Quadrivalent vaccines containing both IBV lineages can reduce B-lineage mismatch, thus reducing the burden of IBV disease.

## Introduction

Seasonal influenza is a public health problem that affects approximately 5–10% of adults and 20–30% of children worldwide each year, and is responsible for significant influenza-related morbidity and mortality, especially in high-risk groups, as identified by the World Health Organization (WHO).^1^

The causative pathogen, influenza viruses, belongs to the ribonucleic acid virus family *Orthomyxoviridae* and can be classified into A, B, and C types.^2^ Contrary to IAV, IBV almost exclusively infects humans,^3^ and is thus not associated with a pandemic risk. Further, IBV is less diverse than IAV as it undergoes slower antigenic drift. However, it is now acknowledged that IBV is common among younger people, can cause epidemics every few years,^4^ and has been associated with a disproportionate number of pediatric influenza deaths.^5^

The most effective way to prevent influenza and its complications is vaccination,^1^ particularly among high-risk individuals (i.e. the elderly, children, people with underlying conditions, and healthcare workers).^6-8^ Current trivalent subunit influenza vaccines (TIVs) are composed of two IAV subtypes (A/H1N1 and A/H3N2) and one IBV lineage (B/Victoria or B/Yamagata). However, any immunological cross-reactivity between the two IBV lineages is unsure, so immunization against one lineage is not expected to provide optimal protection against the other.^9^^,^^10^ Therefore, quadrivalent subunit influenza vaccines (QIVs) and quadrivalent live attenuated influenza vaccines – which contain both IAV subtypes and both IBV lineages – have been developed to reduce the risk of B-lineage mismatch.^11^^,^^12^ However, TIVs are still widely used – in Turkey and internationally – and the decision about which IBV lineage to include in the TIVs each season is based on circulating lineages prior to the start of each influenza season, as assessed using surveillance data. In addition to guiding prevention and treatment strategies with vaccines and antivirals, respectively, surveillance data help in the understanding of influenza epidemiology and virology and, therefore, may help to further forecast and control influenza epidemics.^13^

In this retrospective analysis, we investigated the results of community-based sentinel surveillance of 13 consecutive influenza seasons (2003-2016) in western Turkey, building upon our previous reports.^14-15^ Here, we specifically discuss the data related to the epidemiologic characterization of IBV and the potential implications of these findings on vaccination strategy in Turkey.

## Results

### Specimens collected

**Figure 1.**
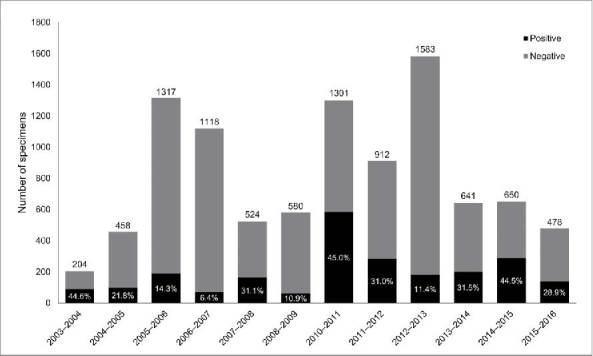
Numbers of specimens tested each season (excluding the pandemic year [2009–2010]) and percentages positive for influenza.

Over the entire study period, 14,429 specimens were collected and tested for influenza viruses, of which 9,766 (67.7%) were detected during seasonal influenza years (weeks 40–20) and 4,663 (32.3%) during the 2009–2010 pandemic year (week 21 of 2009 to week 20 of 2010). Excluding the pandemic year, the number of samples collected each season ranged from 204 in the first season of the preliminary surveillance study (2003–2004) to 1,583 in 2012–2013 (Fig. 1).

#### Positivity of specimens

Over the entire study period, 3,927/14,429 specimens (27.2%) tested positive for influenza viruses – 2,355/9,766 (24.1%) during seasonal influenza years and 1,572/4,663 (33.7%) during the 2009–2010 pandemic year. Excluding the pandemic year, the percentage of positive samples ranged from 6.4% (2006–2007) to 45.0% (2010–2011) (Fig. 1).

#### Pathogen distribution

**Figure 2.**
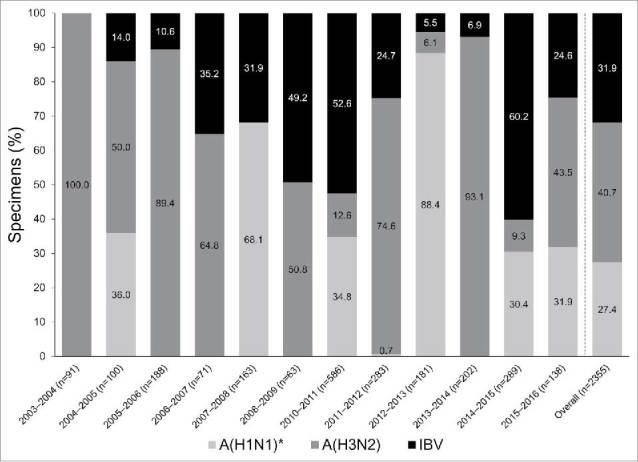
Pathogen distribution, by season and overall (excluding the pandemic year [2009–2010]). *A/H1N1 (2003–2009) or A/H1N1/pdm09 (2010–2016). IBV, influenza B virus.

Excluding the pandemic year (2009–2010), the most commonly reported influenza subtype was A/H3N2 in eight seasons, A/H1N1 in two seasons, and IBV in two seasons (Fig. 2). Overall, excluding the pandemic year, 958 (40.7%) were A/H3N2, 752 (31.9%) were IBV, and 645 (27.4%) were A/H1N1 (Fig. 2). The proportion of IBV among positive samples ranged from 0% (in 2003–2004) to 60.2% (in 2014–2015) (Fig. 2). During the 2009–2010 pandemic, the majority of influenza cases were A/H1N1 (1,545 [98.3%]), with 26 (1.7%) A/H3N2 and one (<0.1%) IBV.

#### Antigenic types in circulation versus those in the vaccine

During and after the 2009–2010 pandemic, A/H1N1 circulating viruses were replaced by A/H1N1/pdm09 viruses. The dominant A/H1N1 or A/H1N1/pdm09 subtypes isolated in our laboratory were found to be compatible with the vaccine strains for all relevant seasons, including during the pandemic year (2009–2010) (Appendix Table S1). However, dominant A/H3N2 viruses only matched the vaccine composition in 3/10 seasons in which antigenic typing was performed (Appendix Table S1).

**Figure 3.**
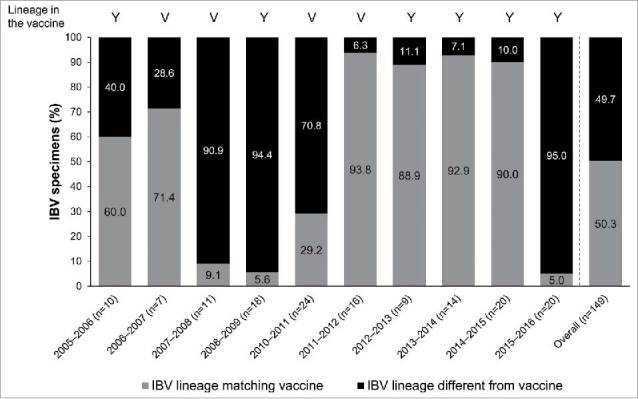
IBV circulation by lineage in Turkey during 2005–2016 (excluding the pandemic year [2009–2010]), by season and overall; and the proportions of IBV specimens tested that matched and mismatched the vaccine IBV lineage. IBV, influenza B virus; V, Victoria; Y, Yamagata.

Excluding the pandemic year (2009–2010), 2003–2004 (no IBV circulating), and 2004–2005 (IBV antigenic type not tested), antigenic characterization revealed that B/Victoria and B/Yamagata lineages co-circulated for three seasons; while during the other seven seasons, only one IBV lineage was predominantly in circulation (Fig. 3). Appendix Table S2 details the antigenic types in the vaccine and in circulation each season. Circulating IBV matched the vaccine composition >50% in six seasons and <50% in four seasons during which antigenic typing was performed (Fig. 3 and Appendix Table S2). During 2005–2016 (excluding the pandemic year [2009–2010]), the average IBV match with the vaccine was 50.3%.

**Figure 4.**
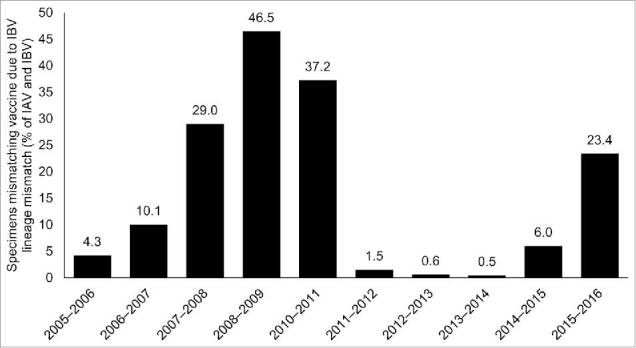
Percentages of all positive specimens (IAV and IBV) estimated* to mismatch the vaccine due to IBV lineage mismatch during 2005–2016 (excluding the pandemic season [2009–2010]). *Based on a subset of IBV samples in which lineage was determined. IAV, influenza A; IBV, influenza B virus.

Combining the data on IAV/IBV distribution (from Fig. 2) and IBV mismatch (from Fig. 3), the percentages of all specimens estimated not to match the vaccine type due to IBV lineage mismatch varied from 0.5% to 46.5% (Fig. 4).

#### Seasonal variability

**Figure 5.**
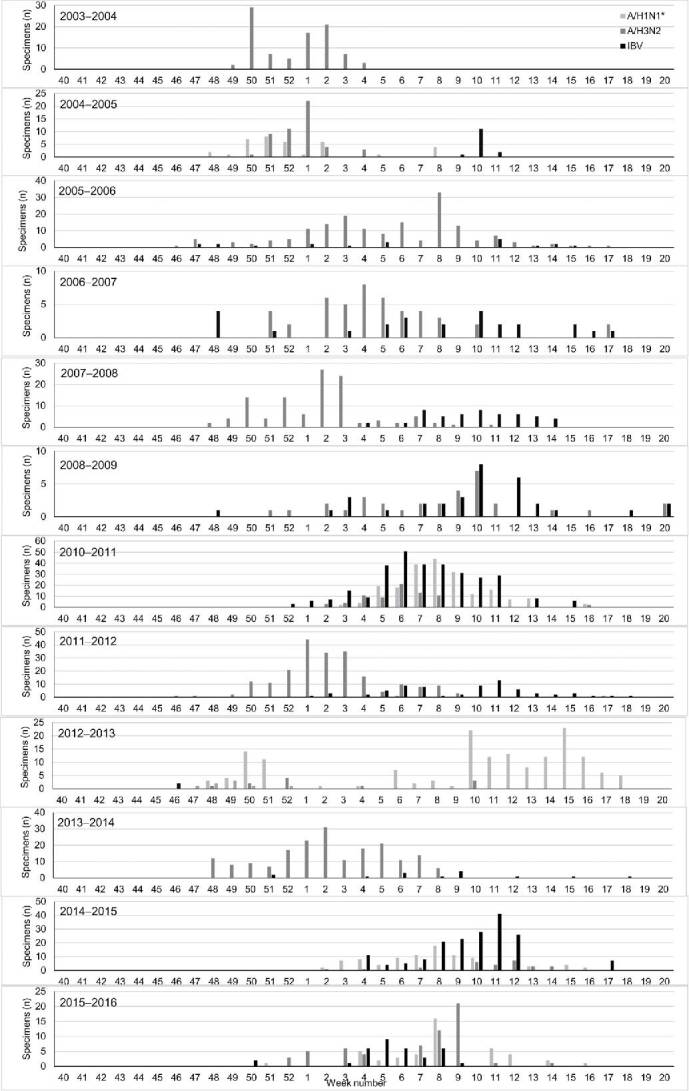
Timing of the influenza activity periods in Turkey (excluding the pandemic year [2009–2010]). Please note that in years with a week 53, these cases were included in week 52. Please also note the varying y-axis scales by season. *A/H1N1 (2003–2009) or A/H1N1/pdm09 (2010–2016). IBV, influenza B virus.

**Figure S1.**
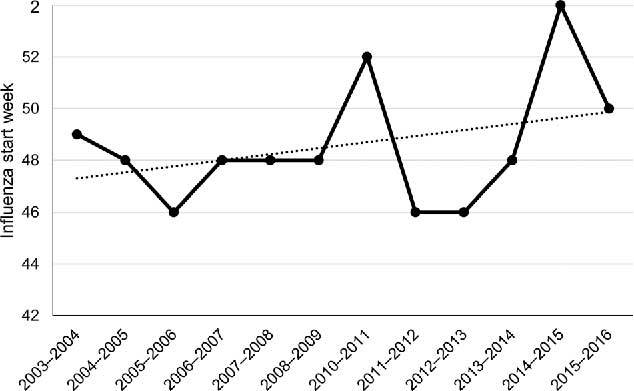
Changes in the start of influenza activity in Turkey (excluding the pandemic year [2009–2010]). The dotted line is an Excel “linear trendline”.

Excluding the pandemic year (2009–2010), IAV cases started to appear any time between weeks 46 and 2, while first IBV cases tended to be reported slightly later in the season (weeks 46–9) (Fig. 5). IBV cases also generally peaked later than IAV cases, although not in every season. During the 2009–2010 pandemic, influenza cases started much earlier than in the interpandemic influenza seasons. Excluding the pandemic year (2009–2010), there was a slight trend towards influenza cases starting later during the season over the course of this study (Appendix Fig. S1).

## Discussion

The number of collected samples and the rates of influenza positivity during 2003–2016 varied substantially by season in western Turkey, from 204 in 2003–2004 to 4,663 in the pandemic year (2009–2010) and from 6.4% in 2006–2007 to 45.0% in 2010–2011, respectively. Differences in positivity may be due to the attitude of the volunteer family physicians and the improved sensitivity of the molecular biology techniques, which we started to use soon after the beginning of the pandemic year (2009–2010). For the standardization of case selection and the collection of samples, we organized a workshop to train the family physicians who participated in the surveillance at the beginning of each influenza season after 2010. Since the 2009–2010 pandemic year, our positivity rate reached 29–45% for each season, except for 2012–2013. This is slightly higher than results from sentinel and non-sentinel surveillance in eastern Turkey, where 20–34% of samples were positive during 2010–2015.^16^

**Figure S2.**
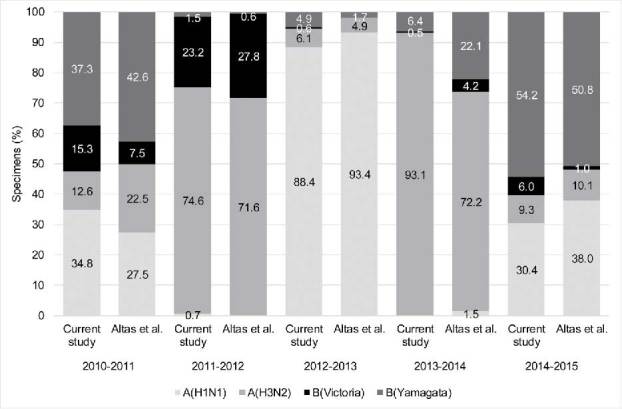
Comparison of the estimated* pathogen splits in the current study (sentinel surveillance in western Turkey) and Altas et al.1 (sentinel and non-sentinel surveillance in eastern Turkey) during the years common to both studies. *Based on subsets of influenza B samples in which lineage was determined.

IAV circulated during all 13 seasons, and accounted for 80.8% of influenza cases overall (including the pandemic year [2009–2010]). Excluding the pandemic year, IBV accounted for 31.9% of samples. The B/Yamagata lineage was the dominant IBV lineage in 6/10 seasons. Overall, our results on IAV subtype and IBV lineage from sentinel surveillance in western Turkey are well aligned with those from sentinel and non-sentinel surveillance in eastern Turkey during the seasons reported in both studies (2010–2015; Appendix Fig. S2).^16^ Our IBV results (19.2% during 2003–2016 including the pandemic year) are also well aligned with those from the Global Influenza B Study,^17^ which reported that IBV accounted for 19.0% of positive samples during various ranges of years since 2000, but this varied by country and years included, from 7.0% in Italy (2002–2012) through 18.9% in Turkey (2006–2011) to 38.3% in Ivory Coast (2007–2012). IBV lineages and vaccine match/mismatch for Turkey are only reported for the non-pandemic seasons during 2007–2011 in the Global Influenza B Study,^17^ and results for these three seasons are aligned with our results.

The proportion of samples containing IBV varied widely by season in the current study, from 0% in 2003–2004 to 60.2% in 2014–2015. This variation is in line with results from other countries. For example, Ambrose and Levin^5^ reported that IBV accounted for 1.0–59.8% of all circulating influenza strains in Europe during 2001–2011; and 0.4–43.6% of those in the US. In Australia during 2000–2011, IBV circulation has been reported to range from 0.8% in 2003 to 63.3% in 2008.^18^ This high variability in IBV circulation may be attributable to variable population immunity and competition between the two co-circulating lineages of IBV, which may differ according to the geographic location where the samples are collected.

In Turkey, the dominant circulating IBV lineage and that in the vaccine were >50% matched in six seasons and <50% matched in four seasons (Fig. 3). This is also similar to findings from other countries. In the study by Ambrose and Levin,^5^ among eight seasons in Europe during 2003–2011, the predominant lineage differed from that contained in the vaccine in four seasons, there was a partial match in two seasons, and a good match in two seasons. They estimated that, overall, 58% of IBV samples were of the lineage not included in the vaccine.^5^ Similarly, in the US, the predominant IBV lineage in circulation did not match that in the vaccine in 5/10 influenza seasons during 2001–2011.^5^ It was estimated that 46% of IBV samples during this period were of the lineage not included in the vaccine.^5^ The Australian data revealed a “low” match of the vaccine strain and the circulating IBV lineage in 4/12 years during 2000–2011, a “medium” match in 3 years, and a “high” or “complete” match in 5 years.^18^

The above-mentioned data show how difficult it is to predict which IBV lineage will dominate in a given season, resulting in predictions only being correct in approximately half of influenza seasons. This type of discordance has important implications for the effectiveness of influenza vaccines, as Cochrane analyses have shown that influenza vaccine efficacy may be reduced when the influenza vaccine strains poorly match the circulating strains.^19-21^ For example, during the 1987–1988 influenza season in Japan, Kanegae *et al.*^22^ investigated a low efficacy of influenza vaccination in school outbreaks and reported the isolation of B/Yamagata/16/88, an antigenically distinct variant of IBV. They noted an attack rate of 83% in a school outbreak in which 100% of the students had been vaccinated with the B/Ibaraki/2/85 strain.^22^

As it is difficult to predict which IBV lineage will be in circulation and as there is limited cross-reactivity between IBV lineages, there is good justification to include both lineages in seasonal influenza vaccines. QIVs have a potential public health impact, i.e. reducing the amount of severe illness (with its associated consequences in the frailest populations [e.g. children, older adults, immunocompromised people, pregnant women). QIVs also have a potential economic impact, i.e. decreased hospitalization and overall healthcare utilization. For these reasons, QIVs are already in use in various countries, and the virus strains recommended for each season's QIVs can be found on the WHO website.^23^

Our investigation concerning the duration and timing of peak influenza activity is an important issue for the development of vaccination policy. Our surveillance data have shown that IBV generally tended to circulate slightly later in the season than IAV. This has also been reported by Finkelman *et al.*^24^ for countries in the northern hemisphere in their analysis of global WHO surveillance data (FluNet).^25^ They reported that IBV peaked approximately 2 weeks after A/H1N1 and approximately 4 weeks after A/H3N2.^24^

However, the most important point about the timing and duration of the influenza epidemics in Turkey is that they started as early as week 46 (early November), peaked as late as week 15 (early April), and could continue into May. As the protection conferred by influenza vaccination may wane beyond 6 months after vaccination,^26^ early vaccination (e.g. in September) could result in waning protection before the season has peaked. Conversely, if vaccination is postponed, influenza could start to circulate before vaccination is complete, which could be even more detrimental. Therefore, influenza surveillance can provide important information to policy makers but, due to seasonal variations, optimal vaccination timing is not an easy decision.

In Turkey, influenza vaccination is recommended for those aged ≥65 years, nursing home residents, individuals with various chronic conditions, and healthcare workers.^27^ However, data indicate that influenza vaccination uptake in Turkey – even in these high-risk groups – is low.^28^ There is currently no preferential or permissive recommendation regarding the use of QIVs over TIVs, either in Turkey or based on the latest WHO recommendations.^23^ Given the burden of IBV disease, the poor predictability of which of the two IBV lineages will be in circulation, and the potentially reduced protection against IBV disease in seasonal epidemics, QIVs that include two IBV lineages would offer additional benefits to reduce the burden of illness.^4^^,^^11^ This study shows that in Turkey, IBV isolation rate and types vary from year to year and also indicate the need for QIV use in the country.

## Limitations

The laboratory in which the study was conducted operating solely in the west part of Turkey, the findings are limited to a restricted geographic area. As with all family physician-based surveillance systems, our results most likely underestimate the real incidence of influenza, as not all patients would have sought care when they had ILI symptoms. Another limitation is the lack of information about the age distribution of cases, overall and by virus type, subtype, and lineage. Also, the limited number of samples that were tested for IBV lineage. Lastly, the changes in the laboratory techniques soon after the beginning of the 2009–2010 pandemic year to more sensitive tests may have introduced disparities in the positivity of samples collected and limit the comparability of the results before and after the 2009–2010 pandemic.

## Conclusions

Surveillance studies, such as this one, are important for determining the effects of influenza on public health, the benefits of use of QIVs and for helping policy makers to implement approaches for reducing influenza burden of disease. Our data show that the length of influenza seasons, as well as the period during which infections occur in Turkey, varies considerably. Influenza seasons started as early as November or as late as January; and ended any time from January to May; with peaks any time from December to March. Further, different influenza types can dominate each season, including IBV; and the IBV lineage contained in the vaccine only matches the lineage in circulation approximately 50% of the time. Given the poor predictability of IBV lineage circulation, the use of QIVs could reduce the likelihood of an IBV-mismatched season, thus reducing the burden of influenza and its associated complications.

## Methods

### Setting

Influenza surveillance in Turkey was initiated as a pilot study in 2003 and is now undertaken by the National Influenza Reference Laboratory, İstanbul Faculty of Medicine. Surveillance in Turkey was launched at a national level by the Ministry of Health in 2004 targeting two National Influenza Centers: one in İstanbul, responsible for the western part of Turkey; the other in Ankara, in charge of the eastern part. To assess the epidemiology and seasonality of influenza in the western part of Turkey, we established a sentinel surveillance system for influenza in five geographically distinct regions (İstanbul, İzmir, Antalya, Bursa, and Edirne provinces; the population living in these 5 provinces was 27.671 million in 2016), using standard case definitions for ILI.^13^ In this study, we examined the sentinel surveillance data obtained by the National Influenza Reference Laboratory in the İstanbul University Faculty of Medicine for the 12 influenza seasons during 2003–2009 and 2010–2016, plus the pandemic year (2009–2010).

Surveillance forms were routinely used during the influenza seasons, and oral informed consent was obtained from patients presenting to their family physician with ILI at the moment of swab taking (in the physician's office) as per national regulations. In accordance with applicable laws and regulations, no clearance of an Ethics Committee is required in Turkey for the retrospective analysis of anonymized data collected within routine influenza surveillance schemes.

### Specimen collection and testing

Nasal/nasopharyngeal swab samples were taken from patients diagnosed with ILI from week 40 of one year to week 20 of the following year during interpandemic influenza seasons. However, during the 2009–2010 pandemic, swabs were taken from week 21 of 2009 through week 20 of 2010.

Samples were collected by volunteer family physicians (10–12 in each of the five regions throughout the study period). Samples were collected in viral transport medium (Virocult® transport culture medium, Medical Wire & Equipment, Corsham, UK) and sent to the laboratory in compliance with cold chain regulations. The samples were tested for the presence of influenza viruses. When the result was positive, the virus type (IAV or IBV) and subtype (for IAV) were determined; IAV subtype and IBV lineage were investigated in a randomly selected subset of IAV and IBV samples and successful subtyping results were shown.

Up until the 2008–2009 season, immune-capture enzyme-linked immunosorbent assay (ELISA)^13^ followed by Madin-Darby canine kidney (MDCK) epithelial cell culture was used for the detection of influenza viruses. Antigenic characterizations were performed by hemagglutination inhibition (HI) assay using reagents supplied by the WHO for the determination of subtype and antigenic characterization.

Soon after the start of the 2009–2010 pandemic, after the pandemic had been declared by the WHO, real-time reverse transcriptase polymerase chain reaction tests (RT-PCR) began to be used for virus detection, typing, subtyping, and determination of IBV lineages.^29^ In summary, all samples were transferred to cryo tubes upon receipt and stored at –80°C if not tested on their arrival date. EZ1 Virus mini kit V2.0 (Catalog number: 955134, Qiagen, Germany) was used for total nucleic acid extraction. For the detection of A/H1N1 and A/H3N2 subtypes and B/Yamagata and B/Victoria lineages, a real-time RT-PCR method was performed using an ABI 7500 platform with Centers for Disease Control and Prevention (CDC) primers and probes according to a CDC-approved protocol.^30–32^

According to the Terms of Reference for National Influenza Centers (NIC),^33^^,^^34^ representative virus isolates were sent to the UK WHO Collaborating Centre for Reference and Research on Influenza of the National Institute for Medical Research. Following the WHO collaborating centers recommendations, the lineage of IBV was only characterized in a random subset of specimens that were sent to the NIC.

## Supplementary Material

KHVI_A_1452577_Supplemental.zip

## Abbreviations

CDC: Centers for Disease Control and Prevention
HI: hemagglutination inhibition
IAV: influenza A virus
IBV: influenza B virus
ILI: influenza-like illness
MDCK: Madin-Darby canine kidney
NIC: National Influenza Centers
QIV: quadrivalent subunit influenza vaccines
RT-PCR: real-time reverse transcriptase polymerase chain reaction
TIV: trivalent subunit influenza vaccine
WHO: World Health Organization

## Appendix

**Table S1.** Dominant (≥90%) antigenic types of IAV in the vaccine and in circulation in Turkey (2003–2016).

	A/H1N1 (2003–2009) or A/H1N1/pdm09 (2009–2016)		A/H3N2	
Season	Vaccine content	Dominant antigenic type in Turkey	Vaccine content	Dominant antigenic type in Turkey
2003–2004	A/New Caledonia/20/99	—	A/Moscow/10/99-like	**A/Fujian/411/02-like**
2004–2005	A/New Caledonia/20/99-like	A/New Caledonia/20/99	A/Fujian/411/2002-like	**A/Netherlands/128/2004**
2005–2006	A/New Caledonia/20/99	—	A/California/7/2004-like	**A/Hong Kong/443/05**
2006–2007	A/New Caledonia/20/99	—	A/Wisconsin/67/2005 or A/Hiroshima/52/2005	A/Wisconsin/67/2005
2007–2008	A/Solomon Islands/3/2006	A/Solomon Islands/3/2006	A/Wisconsin/67/2005 or A/Hiroshima/52/2005	—
2008–2009	A/Brisbane/59/2007	—	A/Brisbane/10/2007	A/Brisbane/10/2007
2009–2010^a^	A/California/7/2009	A/California/7/2009	A/Perth/16/2009-like	Not tested
2010–2011	A/California/7/2009	A/California/7/2009	A/Perth/16/2009	Not tested
2011–2012	A/California/7/2009	A/California/7/2009	A/Perth/16/2009	**A/Victoria/361/2011-like**
2012–2013	A/California/7/2009	A/California/7/2009	A/Victoria/361/2011	**A/Texas/50/2012**
2013–2014	A/California/7/2009	—	A/Texas/50/2012	**A/Victoria/361/2011-like**
2014–2015	A/California/7/2009	A/California/7/2009	A/Texas/50/2012	**A/Victoria/361/2011**
2015–2016	A/California/7/2009	A/California/7/2009	A/Switzerland/9715293/2013	A/Switzerland/9715293/2013

IAV, influenza A virus. Bold text indicates a mismatch of the circulating type with the vaccine type.

a During the pandemic year (2009–2010), samples were collected from week 21 in 2009 through week 20 in 2010.

**Table S2.** Antigenic types/lineages of IBV in the vaccine and in circulation in Turkey (2004–2016).

Season	Vaccine content	Antigenic type(s) in Turkey
2004–2005	B/Shanghai/361/2002-like (Yamagata)	Not tested
2005–2006	B/Shanghai/361/2002-like (Yamagata) or B/Jiangsu/10/2003 (Yamagata)	B/Jiangsu/10/2003 (Yamagata) or B/Florida/7/2005 (Yamagata) (60.0%) B/Malaysia/2506/2004 (**Victoria**) (40.0%)
2006–2007	B/Malaysia/2506/2004-like (Victoria) or B/Ohio/1/2005 (Victoria)	B/Malaysia/2506/2004 (Victoria) (71.4%) B/Florida/4/2006 (**Yamagata**) (28.6%)
2007–2008	B/Malaysia/2506/2004-like (Victoria)	B/Florida/4/2006 (**Yamagata**) (90.9%) B/Malaysia/2506/2004 (Victoria) (9.1%)
2008–2009	B/Florida/7/2006-like (Yamagata)	B/Malaysia/2506/2004 (**Victoria**) (94.4%) B/Florida/4/2006 (Yamagata) (5.6%)
2010–2011	B/Brisbane/60/2008-like (Victoria)	B/Bangladesh/133/07 (**Yamagata**) (70.8%) B/Brisbane/60/2008-like (Victoria) (29.2%)
2011–2012	B/Brisbane/60/2008-like (Victoria)	B/Brisbane/60/2008 (Victoria) (93.8%) B/Florida/4/2006 (**Yamagata**) (6.3%)
2012–2013	B/Wisconsin/1/2010-like (Yamagata)	B/Wisconsin/1/2010-like (Yamagata) (88.9%) B/Brisbane/60/2008 (**Victoria**) (11.1%)
2013–2014	B/Massachusetts/2/2012 (Yamagata)	B/Massachusetts/2/2012 (Yamagata) (92.9%) B/Brisbane/60/2008 (**Victoria**) (7.1%)
2014–2015	B/Massachusetts/2/2012 (Yamagata)	B/Massachusetts/2/2012 (Yamagata) (90.0%) B/Brisbane/60/2008 (**Victoria**) (10.0%)
2015–2016	B/Phuket/3073/2013-like virus (Yamagata)	B/Malaysia/2506/2004 (**Victoria**) (95.0%) B/Massachusetts/2/2012 (Yamagata) (5.0%)

IBV, influenza B virus. Bold text indicates a mismatch between circulating and vaccine lineage.
