# Supplementary material for: Influenza surveillance in Western Turkey in the era of quadrivalent vaccines: A 2003–2016 retrospective analysis
Source: Hum Vaccin Immunother. 2018 Apr 25;14(8):1899–908. doi: 10.1080/21645515.2018.1452577 (PMC6149844; doi:10.1080/21645515.2018.1452577)
Supplement: KHVI_A_1452577_Supplemental.zip [file khvi-14-08-1452577-s001.zip › KHVI_A_1452577_Supplemental Figures.docx]

**Appendix**

**Figure S1.** Changes in the start of influenza activity in Turkey (excluding the pandemic year [2009–2010]). The dotted line is an Excel “linear trendline”.

**Figure S2.** Comparison of the estimated* pathogen splits in the current study (sentinel surveillance in western Turkey) and Altas et al.^1^ (sentinel and non-sentinel surveillance in eastern Turkey) during the years common to both studies.

*Based on subsets of influenza B samples in which lineage was determined.

**Reference**

**1** Altas AB, Bayrakdar F, Korukluoglu G. [Influenza surveillance in five consecutive seasons during post pandemic period: results from National Influenza Center, Turkey]. Mikrobiyol Bul 2016;50:401-17.
