# Supplementary material for: Influenza surveillance in Western Turkey in the era of quadrivalent vaccines: A 2003–2016 retrospective analysis
Source: Hum Vaccin Immunother. 2018 Apr 25;14(8):1899–908. doi: 10.1080/21645515.2018.1452577 (PMC6149844; doi:10.1080/21645515.2018.1452577)
Supplement: KHVI_A_1452577_Supplemental.zip [file khvi-14-08-1452577-s001.zip › KHVI_A_1452577_Supplemental.pptx]

## Slide 1
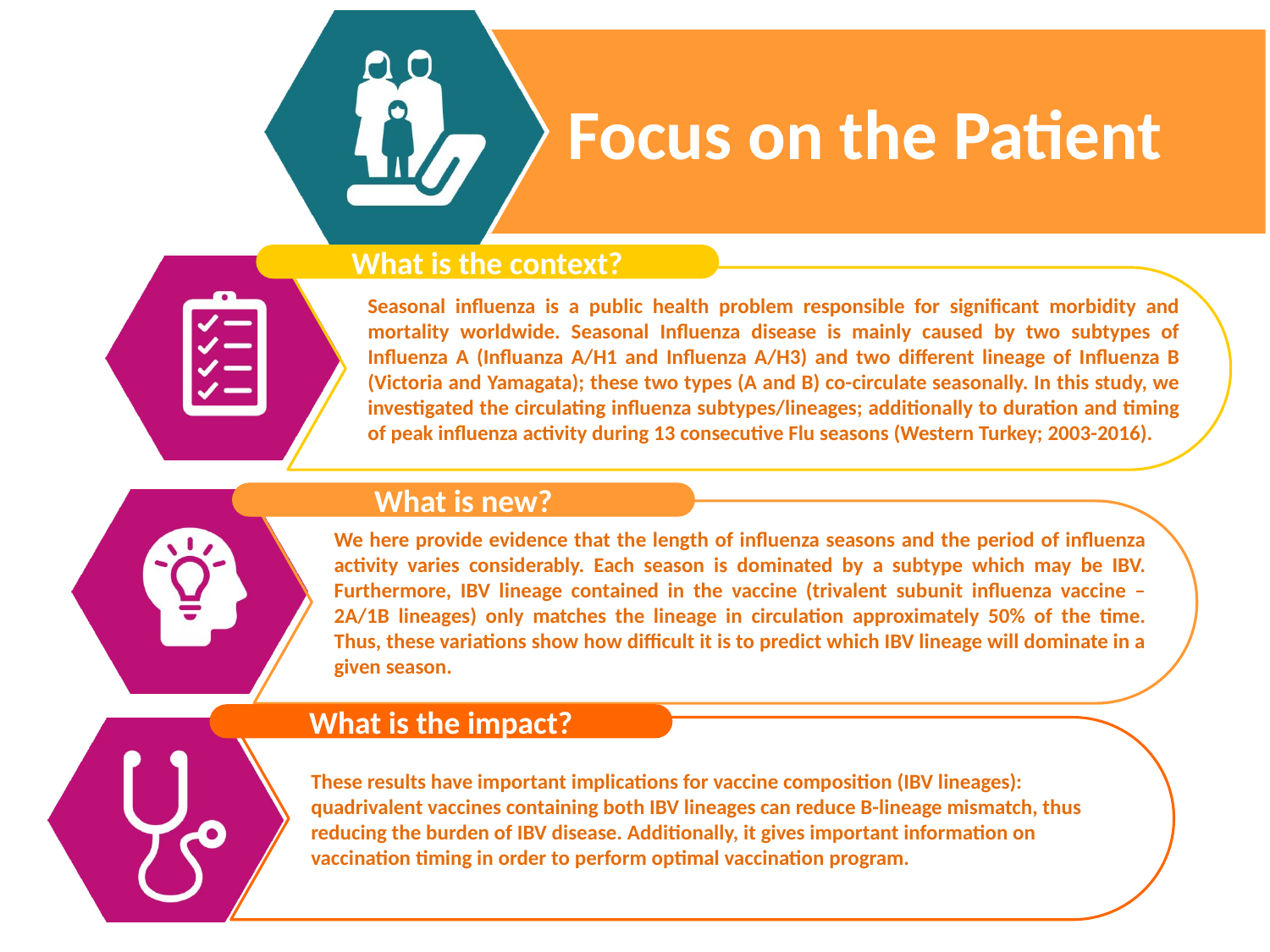

Focus on the Patient
What is the context?
Seasonal influenza is a public health problem responsible for significant morbidity and mortality worldwide. Seasonal Influenza disease is mainly caused by two subtypes of Influenza A (Influanza A/H1 and Influenza A/H3) and two different lineage of Influenza B (Victoria and Yamagata); these two types (A and B) co-circulate seasonally. In this study, we investigated the circulating influenza subtypes/lineages; additionally to duration and timing of peak influenza activity during 13 consecutive Flu seasons (Western Turkey; 2003-2016).
What is new?
We here provide evidence that the length of influenza seasons and the period of influenza activity varies considerably. Each season is dominated by a subtype which may be IBV. Furthermore, IBV lineage contained in the vaccine (trivalent subunit influenza vaccine – 2A/1B lineages) only matches the lineage in circulation approximately 50% of the time. Thus, these variations show how difficult it is to predict which IBV lineage will dominate in a given season.
What is the impact?
These results have important implications for vaccine composition (IBV lineages): quadrivalent vaccines containing both IBV lineages can reduce B-lineage mismatch, thus reducing the burden of IBV disease. Additionally, it gives important information on vaccination timing in order to perform optimal vaccination program.
